# Charting the Normal Development of Structural Brain Connectivity in Utero using Diffusion MRI

**DOI:** 10.1101/2025.09.14.676101

**Published:** 2025-09-15

**Authors:** Davood Karimi, Bo Li, Athena Taymourtash, Camilo Jaimes, Ellen P. Grant, Simon K. Warfield

**Affiliations:** 1 Department of Radiology, Harvard Medical School and Boston Children’s Hospital, Boston, Massachusetts, USA; 2 Electrical Engineering and Computer Science, Massachusetts Institute of Technology, Cambridge, Massachusetts, USA; 3 Department of Radiology, Harvard Medical School and Massachusetts General Hospital, Boston, Massachusetts, USA

## Abstract

Understanding the structural connectivity of the human brain during fetal life is critical for uncovering the early foundations of neural function and vulnerability to developmental disorders. Diffusion-weighted MRI (dMRI) enables non-invasive mapping of white matter pathways and construction of the brain’s structural connectome, but its application to the fetal brain has been limited by data scarcity and technical difficulties in analyzing fetal dMRI data. Here, we present the largest study to date of in utero brain connectivity, analyzing high-quality dMRI data from 198 fetuses between 22 and 37 gestational weeks, from the Developing Human Connectome Project. We employed advanced fetal-specific tools for brain segmentation, parcellation, and tractography. For connection weighting, we relied on the notion of fiber bundle capacity. We reconstructed individual structural connectomes and characterized the developmental trajectories. Graph-theoretical analysis revealed consistent increases in integration and segregation metrics over gestation, alongside stable small-world properties. Bootstrapping confirmed the robustness of nodal and edge-wise developmental patterns, and a sigmoid growth model identified a narrow time window (around 27.5–30.5 weeks) of rapid connectivity strengthening. Furthermore, we proposed a novel method for constructing age-specific connectome templates based on aggregation of individual subject connectomes. Our analysis shows that this approach is superior to spatial alignment and averaging of the data in image space, with the connectome templates preserving individual topology and supporting accurate age prediction. Together, our findings provide a reasonable normative map of fetal brain structural connectivity and establish a foundation for future studies of atypical development and early indicators of neurological risk.

## 1 Introduction

Understanding the brain’s structural connectivity is a fundamental goal in neuroscience [1–4]. The structural connectome underpins and supports all brain activities. Moreover, it can influence and be influenced by the brain’s response to diseases. Diffusion-weighted MRI (dMRI) is a unique non-invasive tool for studying the brain’s structural connectivity [5–8]. The dMRI signal can be used to estimate the local orientation of white matter fibers in the brain. Building on this local information, tractography algorithms can be applied to trace virtual streamlines connecting predefined brain regions. These streamlines, typically weighted based on measures of tissue microstructure integrity or fiber bundle capacity, determine the strength of connections between the regions to compute a structural connectome [9–11]. The connectome may be regarded as a mathematical *graph*, where regions represent the graph nodes and the connections represent the edges. Graph-theoretic metrics can subsequently be computed to quantitatively analyze the connectome [4, 6, 12].

Although dMRI-based connectivity analysis suffers from technical challenges, constant methodological advancements have improved its accuracy and reproducibility [11, 13, 14]. Moreover, our understanding of the capabilities and limitations of this method have greatly improved and we can more reliably interpret the quantitative results [15–18]. There have been numerous efforts to study the brain development, aging, and degeneration based on structural connectivity [10, 19–22]. Extensive research on adult brains has shown that neurological diseases such as epilepsy, dementia, Alzheimer’s disease, schizophrenia, ADHD, and multiple sclerosis are strongly associated with disruptions in the structural connectome [23–34]. These findings suggest that structural connectivity plays a central role in how the brain is affected by the diseases and how it responds to them.

However, prior works on structural connectivity have almost exclusively focused on postnatal and adult brains. By comparison, the fetal brain has received far less attention [35–42]. This has been, in part, due to the limited availability of fetal dMRI data. While there have been large publicly available datasets of pediatric and adult brain dMRI, until recently similar datasets for fetal brain have been lacking. Fetal dMRI also suffers from lower signal to noise ratio, unpredictable motion, and typically shorter scan times [7, 43, 44]. Moreover, due to the incomplete myelination and relatively higher water content, the fetal brain tissue microstructure is different from that of adult brain [45, 46]. Additionally, the fetal brain undergoes rapid development within a short period. Tissue microstructure undergoes significant changes during gestation as white matter tracts emerge, develop, and myelinate at varying time points and with different rates throughout this period [39,43]. Thus, analyzing the fetal brain dMRI data requires dedicated computational methods that are not widely available. As a result, little is known about the development of the structural connectome in the fetal stage.

This represents a significant gap in knowledge as the fetal period is a dynamic and critical stage in brain development [3,35,47–50]. Neurogenesis, neural migration, synapse formation, and axonal growth all begin before birth, forming the brain’s microstructure and laying the foundations of its networks [3,36,51–59]. It is well known that adult-like brain structures and a highly organized connectome develop in utero [43, 55, 58, 60–63]. There is also growing evidence that the structural connectivity of the fetal brain can be disrupted by diseases and environmental factors, potentially resulting in lifelong neurodevelopmental and psychiatric disorders [48, 64–66]. For example, prenatal exposure to maternal stress, congenital heart disease, and brain malformations can alter the structural connectivity of the brain in utero [38, 67–75]. Therefore, a quantitative assessment of the structural connectome during the fetal period can deepen our understanding of the formation and development of the brain networks and may facilitate the identification of high-risk fetuses [7, 37, 74, 76, 77].

Prior studies on the fetal brain are very few in number and suffer from several limitations [38, 56, 74]. First, they have analyzed small populations and narrow gestational age ranges. Moreover, they have often relied on elementary and simplistic computational methods. For example, they have inferred the local orientation of major fibers using a diffusion tensor model, which cannot resolve crossing fibers. Most prior works have used rudimentary measures of connection strength, e.g., the number of streamlines, which are known to be inadequate and biased [10, 11, 13], and they have not used microstructure-informed filtering to improve the tractography results. Furthermore, due to reliance on standard computational methods originally devised for adult brains, they are prone to suboptimal and erroneous tractography results [78,79].

In this work we aimed to chart the development of structural connectivity in the fetal period. We used the recent release of the developing Human Connectome Project dataset with dMRI scans from approximately 250 fetuses between 22 and 37 gestational weeks [80]. We leveraged fetal-specific methods for tissue segmentation, parcellation, and tractography. We computed the structural connectome for each fetus and determined how the connectivity metrics changed as a function of gestational age. We assessed the reproducibility of the results using bootstrapping methods. Moreover, we developed two methods to compute representative connectomes for each gestational week. One of these methods was based on precise spatial alignment and averaging of the imaging data, whereas the other method was based on aggregating the connectomes of individual subjects. We applied these methods to compute age-specific connectomes that depicted the temporal changes in structural connectivity between 22 and 37 gestational weeks. Using these atlases, we analyzed the major developmental patterns in intra- and inter-lobe connectivity within and across brain hemispheres. Overall, this study offers new insights into the development of the structural connectome in its earliest stages and provides benchmarks for the normative development of structural connectivity in the fetal period.

## 2 Methods

### 2.1 Data and Preprocessing

This study used the fetal data from the Developing Human Connectome Project. The dataset includes 279 scans from 250 fetuses. We only considered scans with both structural (T2-weighted) and dMRI images, resulting in 259 MRI scan data from 239 fetuses. The dMRI data have been processed with the SHARD pipeline [80]. We did not perform any further preprocessing on the data, except for registering the dMRI data to the T2-weighted image for each fetus and resampling both dMRI and T2-weighted images to an isotropic voxel size of 1 mm. Quality control was performed by an expert with six years of experience in fetal dMRI analysis via visualization of diffusion tensor maps, fiber orientation maps, and whole-brain tractograms in three orthogonal views. As a result, a total of 198 scans, each from a different fetus, were selected for structural connectivity analysis in this work.

### 2.2 Fiber Orientation Estimation and Tractography

Each dMRI scan in this dataset consists of measurements with b=0 (n=15), b=400 (n=46), and b=1000 (n=80). As also reported in prior works [45], we found that standard multi-shell multi-tissue constrained spherical deconvolution [81] was unable to correctly estimate the partial volume fractions for white matter, gray matter, and cerebrospinal fluid (CSF) in the fetal brain. Therefore, to estimate the fiber orientation density (FOD), we used the single-shell constrained spherical deconvolution (CSD) [82], applied to the b=1000 shell data. We also computed the diffusion tensor images using the b=1000 data, which consistently produced visually superior results than with the b=400 shell data.

We applied a deep learning method [83], using the diffusion tensor image as input, to compute the tissue segmentation. Segmentation labels included white matter, cortical gray matter, subcortical gray matter, and CSF. For the cortical plate, we found that the segmentation from the T2-weighted image resulted in more accurate delineation of cortical foldings. Therefore, we combined the segmentation of the cortical plate from the T2-weighted image with segmentation of the white matter, sub-cortical gray matter, and CSF from dMRI. The same deep learning method [83] was also applied to compute a parcellation of the gray matter to define the nodes for the structural connectome.

We performed anatomically constrained tractography using the iFOD2 algorithm [84] to propagate the streamlines, with a well-tested pipeline optimized for tractography of the fetal brain in the second and third trimesters [78]. Streamline seeding was performed on all white matter voxels and also on the boundary between white matter and gray matter. We noticed that some of the tracts were more fully reconstructed by using the diffusion tensor as input while others were better reconstructed using the FOD computed with CSD. Moreover, a single setting of the angle threshold and FOD cutoff threshold was not adequate for computing all tracts. Therefore, we followed an ensemble tractography approach by choosing ten different combinations of fiber orientation inputs and the angle / FOD cutoff thresholds. Specifically, we used angle thresholds in the range [10°, 35°] and FOD cutoff thresholds in the range [10^−5^, 10^−3^]. We computed 10^5^ streamlines with each setting, for a total of 10^6^ streamlines for each brain. For each brain, we computed the structural connectome using the approach proposed in [9, 85]. This approach uses the SIFT2 algorithm [86] to properly weigh the contribution of the streamlines to the structural connectivity strength.

### 2.3 Connectivity Metrics and Statistical Analysis

From the structural connectome of each fetus, we computed standard graph-theoretic descriptors of structural connectivity including global efficiency (GE), local efficiency (LE), characteristic path length (CPL), clustering coefficient (CC), and small-world index (SWI) [12]. We computed the strength of each node as the sum of connection strengths to all its neighbors.

We performed linear regression analyses to determine whether the connectivity metrics, the connection strength of each node, and the strength of each connectome edge (i.e., connection between each node pair) changed significantly with gestational age. For nodal strength, we additionally explored a sigmoid model, as suggested in a recent study on functional connectivity of the fetal brain [87]. This model is defined as

(1) $$f(t)=b+\frac{L}{1+\text{exp}\left[-k\left(t-t_{0}\right)\right]},$$

where $t_{0}$ denotes the inflection point of the model, i.e., the point of fastest increase in the connectivity strength. We used the Levenberg-Marquardt method to fit this function to data. We used the adjusted R-squared to compare the sigmoid model fit with the linear fit.

We analyzed the reproducibility of the connectomics computations via random data sampling in two ways.

1. From each scan, we used two mutually exclusive subsets of the measurements, each with seven b=0 measurements, 23 b=400 measurements, and 40 b=1000 measurements. We reconstructed the connectome and computed the connectivity metrics using each subset. We assessed the reproducibility of the quantitative connectivity analysis by comparing the intra-subject and inter-subject differences and variability in the connectome and connectivity metrics.
2. We randomly selected subsets of the subjects, performed the regression analyses described above using each subset, and analyzed the reproducibility of the results.

### 2.4 Computation of Age-specific Connectomes

There is usually significant inter-subject variability in neuroimaging data. Atlases are used to characterize the population averages and represent typical brain development. We aimed to compute a representative structural connectome for each gestational week between 22 and 37, at one-week intervals. Specifically, we would like to leverage the data from individual fetuses around each gestational week to compute a connectome that represents the typical connectome for that gestational week. We refer to these as “age-specific connectomes”. We developed two different approaches for this purpose, one approach based on aggregating the connectomes computed for each individual fetus and another approach based on data averaging in the image space. These two approaches are described in detail below.

#### 2.4.1 Approach 1: Connectome aggregation

In this approach, we reconstructed an age-specific connectome for each gestational week between 22 and 37 based on the connectomes computed separately for individual fetuses. Each connectome (i.e., weighted adjacency matrix) is represented as a square symmetric matrix in ${\mathbb{R}}^{d\times d}$, where d is the number of connectome nodes. Let us denote the set of connectomes for all N individual fetuses with ${\left\{C_{s}^{i}\right\}}_{i=1:N}$, where $C_{s}^{i}$ is the *subject* connectome for fetus i. Our goal is to compute a set of representative *template* connectomes ${\left\{C_{T}^{j}\right\}}_{j=1:M}$, one for each of M gestational weeks. We consider three desiderata for the connectome template.

##### Desideratum 1- Representation

We would like the representative connectome for each week to closely represent the connectome for individual fetuses with a similar gestational age. To quantify the difference between the template and individual connectomes, we use the graph edit distance (GED). The GED between two graphs $C^{1}$ and $C^{2}$ is computed as the (minimum) cost of a set of edit operations to transform one graph into another [88]. These edit operations include insertion, deletion, and substitution of either a node or an edge. In general, for arbitrary graphs, computing the exact GED is NP-hard. However, in this application all connectomes have the same fixed nodes. Therefore, GED only includes the costs associated with insertion, deletion, or substitution of edges. If we quantify the cost of each edge edit operation (i.e., edge insertion, deletion, or substitution) as the difference in edge weights due to that operation, GED will be the $\ell_{1}$ distance between the connectomes $\text{GED}\left(C^{1},C^{2}\right)=\left|C^{1}-C^{2}\right|$. Therefore, we define the “representation loss” as ${ℒ}_{\text{repr}\;}\left(C_{T}|C_{s}\right)=\underset{i=1:N}{\sum }\underset{j=1:M}{\sum }G\left(t^{i},t^{j}\right)\left|C_{s}^{i}-C_{T}^{j}\right|$. In this equation, $t^{i}$ is the gestational age of subject i and $t^{j}$ is that of the representative connectome $C_{T}^{j}$. Moreover, $G\left(t^{i},t^{j}\right)=\text{exp}\left(-{\left(t^{i}-t^{j}\right)}^{2}/2\sigma^{2}\right)$ is a Gaussian kernel that encourages similarity of age-specific representative connectomes to the individual fetuses that are closest in gestational age. Similar to prior fetal imaging studies [43, 89], we set σ=1.

##### Desideratum 2- Temporal consistency.

We would also like to encourage temporal consistency in the computed age-specific connectomes. To this end, we penalize the GED between the templates with close gestational weeks. Similar to the representation loss above, we use a Gaussian kernel. Hence, our proposed loss term to encourage temporal consistency of the age-specific connectomes is ${ℒ}_{\text{cons}}\left(C_{T}\right)=\underset{j_{1}=1:M}{\sum }\underset{j_{2}=1:M}{\sum }G\left(t^{j_{1}},t^{j_{2}}\right)\left|C_{T}^{j_{1}}-C_{T}^{j_{2}}\right|$.

##### Desideratum 3- Preservation of long connections.

Another consideration is proportional representation/preservation of long-range connections. It is known that short-range connections are more likely to be shared across different structural connectomes [90, 91]. As a result, standard methods for combining connectomes based on connection consensus are biased towards preserving more of the short-range connections and discarding the long-range connections [90]. To compensate for this inherent bias, longer connections should be preferentially preserved in order to ensure that the template/average connectome is similar to the individual subject connectomes in terms of the distribution of connection lengths. One possible approach is to use a variable consensus threshold to preferentially preserve longer connections [90]. In this work, we use the Jensen-Shannon (JS) Divergence to penalize the difference between distance-wise distribution of the connectome weights. Specifically, for an arbitrary pair of connectomes $C^{1}$ and $C^{2}$, we compute the loss function ${ℒ}_{JS}\left(C^{1},C^{2}\right)=D_{JS}\left(p\left(C^{1}\right),p\left(C^{2}\right)\right)$. Here, $p\left(C^{i}\right)$ denotes the distance-wise distribution of the connection weights for connectome $C^{i}$, and $D_{JS}$ is computed based on the Kullback Leibler Divergence as $D_{JS}(p,q)=D_{KL}(p\|(p+q)/2)+D_{KL}(q\|(p+q)/2)$. We approximate p(.) using histograms with 20 bins. Moreover, we include a Gaussian weighting based on a similar logic as presented above for the other two loss terms. Hence, our proposed loss term is ${ℒ}_{\text{dist}\;}\left(C_{T}|C_{s}\right)=\underset{i=1:N}{\sum }\underset{j=1:M}{\sum }G\left(t^{i},t^{j}\right){ℒ}_{JS}\left(C_{s}^{i},C_{T}^{J}\right)$.

Based on these three desiderata, we propose to compute the connectome templates by solving the following optimization problem.


(2) $$\underset{C_{T}}{\text{minimize}}\;{ℒ}_{\text{repr}\;}\left(C_{T}|C_{s}\right)+\lambda_{\text{cons}\;}{ℒ}_{\text{cons}\;}\left(C_{T}\right)+\lambda_{\text{dist}\;}{ℒ}_{\text{dist}}\left(C_{T}|C_{s}\right)$$

Note that in order to simplify the presentation, as in our descriptions above, in Equation (2) we have not shown the indices. To be clear, in Equation (2), $C_{T}\equiv {\left\{C_{T}^{j}\right\}}_{j=1:M}$ and $C_{s}\equiv {\left\{C_{s}^{i}\right\}}_{i=1:N}$. We solve a single optimization problem summarized in Equation (2) to recover age-specific connectomes for all M gestational weeks.

As the initial estimate to start the optimization in Equation (2), we computed $C_{T}$ via simple averaging of the connectomes in each gestational week. For example, for gestational age of 30 weeks we averaged the connectomes for fetuses between 29.5 and 30.5 weeks. We then iteratively optimized $C_{T}$ using stochastic gradient descent (SGD). In each optimization iteration we chose one of the subject connectomes ${\left\{C_{S}^{i}\right\}}_{i=1:N}$ at random and optimized $C_{T}$ using SGD. We found that a small learning rate of 10^−5^ was necessary to ensure stable convergence of the optimization. Nonetheless, given the small size of the problem, the optimization was completed within a few seconds.

#### 2.4.2 Approach 2: Data averaging in the image space

**Figure 1:**
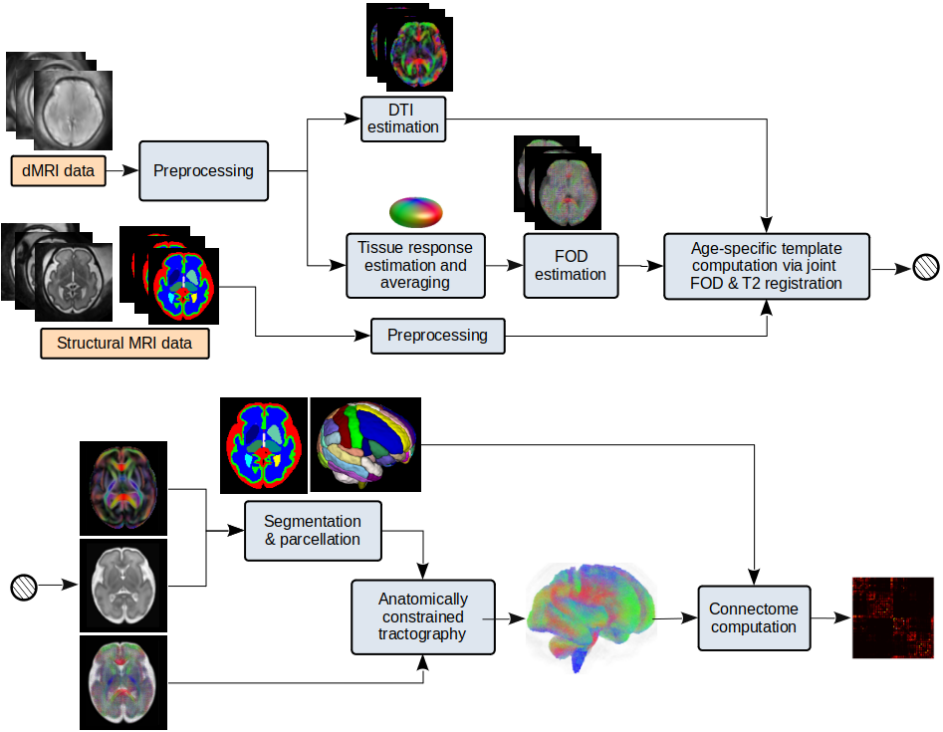
Image-space data averaging for computing a gestational age-specific connectome for each gestational week. Structural MRI and dMRI data from fetuses within a narrow window around the target gestational age are used as input to the pipeline. DTI and FOD maps are computed and precisely aligned using registration methods [92–94]. Segmentation/parcellation maps are computed either by fusion of subject-level data [95] or with deep learning methods [83].

This approach is based on the precise alignment and “averaging” of the imaging data from subjects with a similar gestational age. Different steps of this approach are summarized in Figure 1. The main steps in this approach are summarized below.

1. Preprocess the structural MRI and dMRI data for each subject separately and register the dMRI to the structural MRI.
2. Divide all subjects into gestational age groups. In this work, we assigned each subject only to its closest gestational week. Specifically, all fetuses with a gestational age in the range [t-0.5,t+0.5) were assigned to the gestational week of t. For each gestational age group:
  - Compute the tissue response function for each subject [96] and average them to obtain a mean response function for the age group. Use this average response function to compute the FOD map for each subject using spherical deconvolution [82]. Compute a joint structural MRI and dMRI atlas for the age group via registration of data from all subjects within the age group. This is performed using both T2-weighted images and FOD maps to align the subject imaging data [93,97]. The output of this step includes T2-weighted MRI, diffusion tensor, and FOD atlases for the gestational age group.
  - Compute the tissue segmentation and cortex parcellation with a deep learning method [83] using the diffusion tensor atlas as input. Use the cortical plate segmentation obtained from the T2-weighted atlas to refine the tissue segmentation and parcellation maps.
  - Compute the whole-brain tractogram based on the FOD and diffusion tensor atlases and tissue segmentation using our method described in [78]. We used an ensemble tractography approach similar to that described for individual fetuses in Section 2.2. Finally, compute the connectome.

We compared the above approaches with two existing techniques that are based on connectome aggregation [90, 91]: (1) Consensus-based thresholding, where the threshold is set such that the binary density of the connectome template is equal to the average binary density of the subject connectomes within the gestational age group. (2) Distance-preserved averaging, where different thresholds are used for different connection lengths such that the distribution of the connection lengths in the connectome template is close to that of the subjects. Detailed descriptions of these methods are presented in [90].

We assessed the different methods by comparing the age-specific representative connectomes to the connectomes of individual subjects in two ways.

1. First, we compared scalar connectivity metrics between the representative connectomes and individual subject connectomes of the same age group.
2. Secondly, we evaluated the similarity of each individual fetal subject connectome to the connectome template of the corresponding age using a more sophisticated classification method similar to that proposed in [98]. In this approach each connectome was represented as a feature vector comprising all edge weights and eight topological descriptors. Pairwise differences between individual subject connectomes and template connectomes were summarized as the $\ell_{1}$, $\ell_{2}$, and $\ell_{\infty }$ norms of the differences in their feature vectors. The resulting vector of size three is regarded as the feature vector that is used by a binary support vector machine classifier. The classifier is trained to predict whether a fetus and a template belonged to the same gestational age.

## 3 Results and Discussion

### 3.1 Development of the Structural Connectivity with Gestational Age

**Figure 2:**
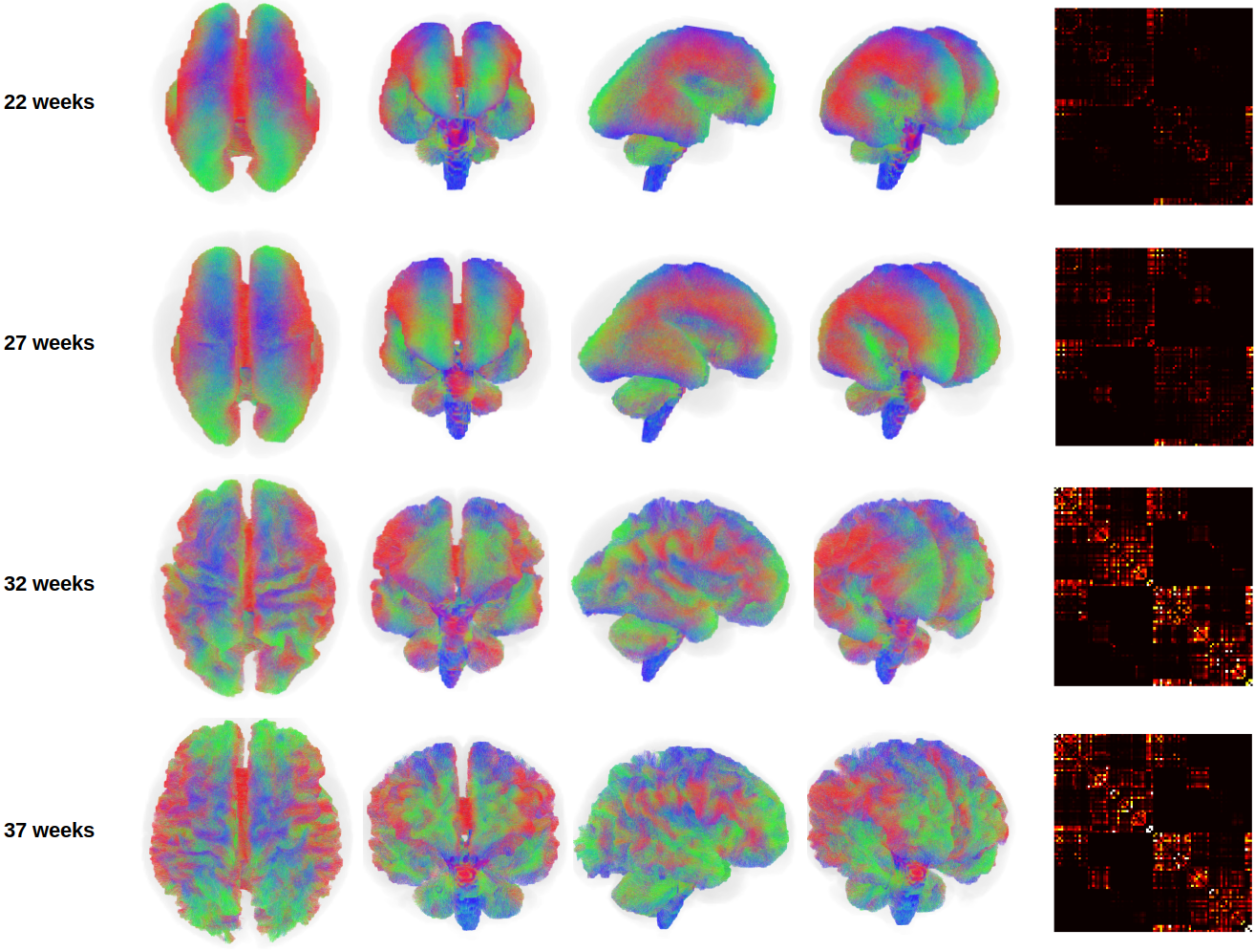
Different views of the whole brain tractograms, and the connectome matrices for four fetuses at different gestational ages. The tractograms for the lower gestational ages have been enlarged to the size of the higher gestational ages for better visualization. The connectomes for all gestational weeks have been shown with the same display intensity window of [0,5].

**Figure 3:**
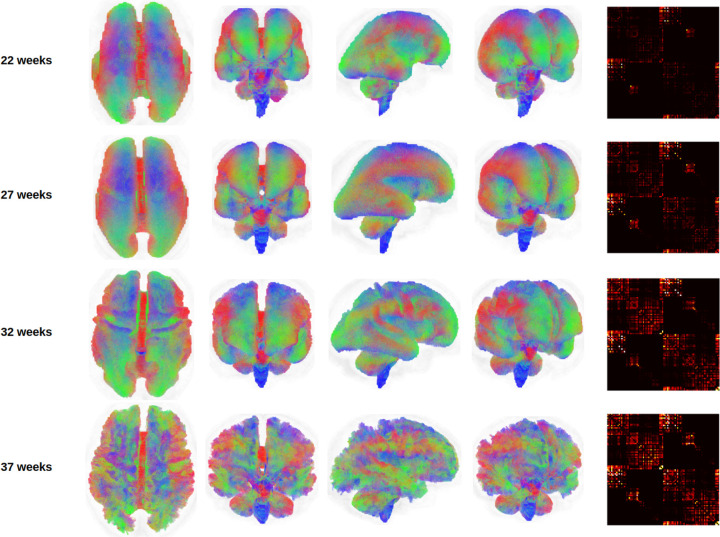
Tractograms and connectomes computed via data averaging in the image space, as described in Section 2.4.2, for four different gestational ages. The tractograms for the lower gestational ages have been enlarged to the size of the higher gestational ages for better visualization. The connectomes have been shown with the same display intensity window of [0,5].

Figure 2 illustrates whole-brain tractograms and structural connectome matrices for four fetuses at 22, 27, 32, and 37 gestational weeks. All computational steps were visually inspected by an expert with six years of experience in fetal dMRI analysis. This quality control included evaluation of color-coded fractional anisotropy maps derived from diffusion tensor imaging, fiber orientation distribution (FOD) maps computed using constrained spherical deconvolution (CSD), as well as tissue segmentation, parcellation maps, and final whole-brain tractograms. Data samples exhibiting lower image quality or clear computational errors were excluded, with the most frequent reasons being suboptimal FOD estimation or segmentation artifacts leading to implausible tractography. For comparison, Figure 3 presents age-specific average tractograms and connectomes, generated via data averaging in the image space as described in Section 2.4.2 and Figure 1.

#### 3.1.1 Changes in the global network metrics

**Figure 4:**
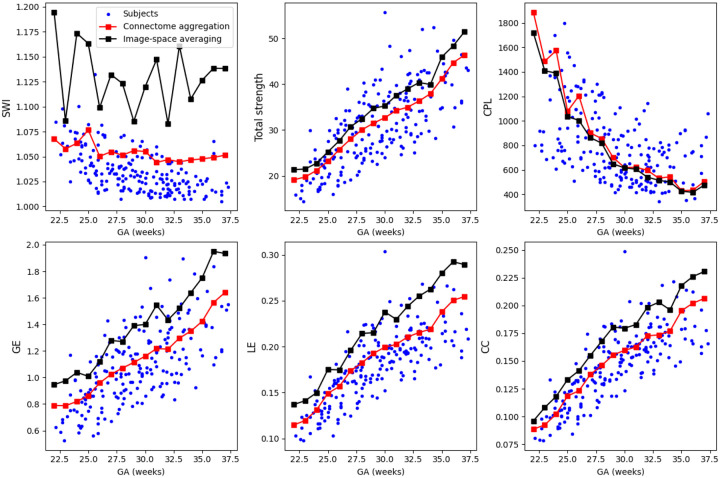
Plots of summary network metrics as a function of gestational age. Each plot shows the values for the subjects as blue dots and the connectome templates as red or black squares connected with line segments.

Figure 4 shows the developmental trajectories of several key graph-theoretical metrics – global efficiency (GE), local efficiency (LE), characteristic path length (CPL), clustering coefficient (CC), small-world index (SWI), and total network strength (defined as the sum of nodal strengths across all regions) – for individual fetuses between 22 and 37 gestational weeks. In addition to individual trajectories, the figure includes these metrics computed from two connectome templates, constructed using the methods described in Section 2.4.

Overall, the plots reveal consistent and developmentally meaningful trends. Global efficiency steadily increases while characteristic path length decreases, indicating a progressive enhancement in network integration as gestation advances. These changes suggest that the fetal brain becomes increasingly optimized for long-range communication, enabling more efficient transfer of information between anatomically distant regions. Simultaneously, both local efficiency and the clustering coefficient increase, reflecting the gradual reinforcement of short-range, locally interconnected sub-networks that support specialized functional processing. The concurrent rise in integration (GE↑, CPL↓) and segregation (LE↑, CC↑) underscores the emergence of a small–world topology, an architectural hallmark of mature and efficient neural systems that balance local specialization with global integration.

A small-world configuration refers to a network architecture that supports both local specialization and global integration with high efficiency. It is defined by (1) a high clustering coefficient, reflecting densely interconnected neighboring brain regions that support local processing, and (2) a short characteristic path length, enabling rapid communication across distant brain areas [99]. In our analysis, the small-world index (SWI) remains relatively stable across gestational ages, maintaining values in the range of 1.05–1.10. This stability occurs despite simultaneous increases in both global efficiency and clustering coefficient, suggesting that the relative balance between integration and segregation is preserved during late gestation. This observation implies that the fetal brain sustains a small–world topology throughout this period, supporting the hypothesis that small–world properties are intrinsic and robust features of neural systems emerging early in development and maintained as the brain undergoes structural maturation. The preserved SWI may also indicate that the underlying developmental mechanisms scale proportionally, affecting both local and global organization in a coordinated manner.

Total network strength also shows a robust and monotonic increase with gestational age, reflecting a continuous rise in the overall connectivity of the brain. In this study, connection strength was quantified using the concept of fiber bundle capacity [9], a biologically informed metric that provides a more accurate estimate of white matter integrity and connection density than traditional measures such as streamline count. The steady increase in total strength likely mirrors the progressive development of white matter tracts, as axonal growth, myelination, and axon packing density all intensify throughout late gestation. Collectively, these findings highlight a phase of rapid and coordinated reorganization of the fetal brain’s structural network, marked by increasing complexity, stronger integration, and the gradual emergence of modular substructures. This evolving architecture is well aligned with the brain’s preparation for functional specialization and the demands of extra-uterine life.

Interestingly, the developmental curves computed from the connectome aggregation-based atlas closely mirror the average trends observed in the individual fetuses. This suggests that this method preserves the statistical and topological features of the underlying population well. In contrast, the connectome metrics computed from the template constructed via image-space averaging show notable deviations in the absolute values compared to those of individual subjects. Although the general trends with gestational age remain qualitatively consistent, the level shifts suggest that the averaging process in the image space introduces systematic alterations in the underlying connectivity patterns. This likely reflects the fact that when diffusion data from multiple fetuses (of similar gestational age) are aligned and averaged in the image space, the resulting atlas represents a spatial and microstructural average that smooths out some of the individual variability in structural connectivity. While such an atlas may offer a robust and anatomically coherent reference for the average brain at a given developmental stage, capturing shared morphological and diffusion features, it inevitably alters subject–specific details that contribute to individual connectome topology. Hence, the connectome templates obtained with this method may underrepresent inter–individual variability and distort the strength and specificity of white matter pathways. In particular, directional coherence, peak fiber density, or sharp anisotropic features that impact quantitative connectivity results may be blunted. These changes can systematically influence network topology, leading to systematic biases in graph–theoretical metric values that diverge from those observed in individual data.

#### 3.1.2 Changes in the nodal and edge strength with gestational age

**Figure 5:**
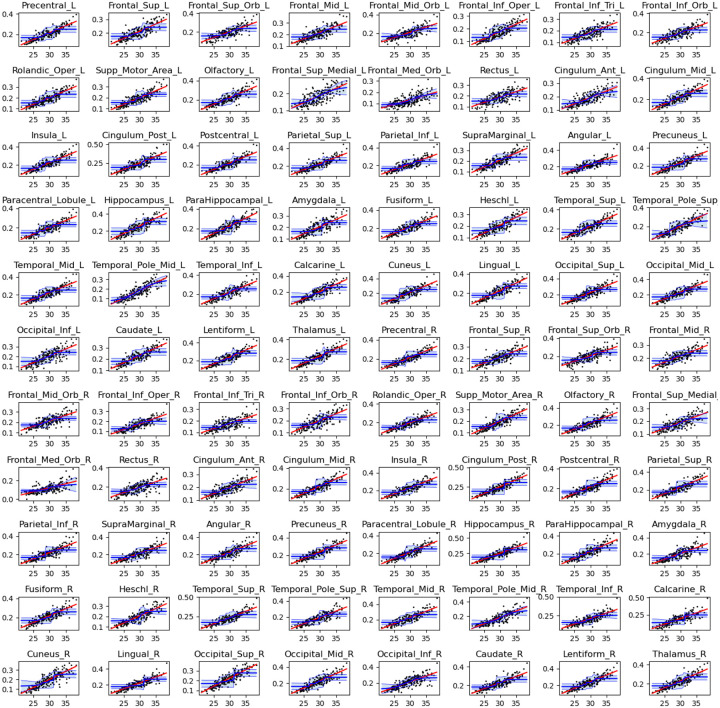
Connection strength for each of the 88 brain nodes as a function of gestational age. The black points represent data for individual fetuses. The red line is the linear fit. The blue lines and shade show the mean ± two standard deviations for the sigmoid fit.

Figure 5 displays scatter plots of nodal strength versus gestational age for each of the 88 brain regions included in the connectome. These plots illustrate a consistent developmental pattern across all nodes: nodal strength increases significantly as a function of gestational age. Linear regression analysis confirmed a statistically significant positive slope for all nodes, even under stringent control for multiple comparisons. Specifically, we used a significance threshold of α=0.01 and applied Bonferroni correction to account for testing across 88 regions.

To capture potential nonlinear patterns in the development of nodal strength, we fit a sigmoid growth model to the data for each brain region. Given the non-convex nature of sigmoid fitting, we employed a bootstrapped approach: the model was fit 1,000 times for each node, each iteration using a randomly selected 75% subset of the fetal subjects (without replacement). Figure 5 displays the mean of these fitted curves along with the ±2 standard deviation confidence bounds derived across iterations. Model comparison revealed that, for most nodes, the sigmoid model outperformed a linear model in terms of adjusted R-squared, indicating a better fit to the observed data. These findings suggest that nodal strength increases in a nonlinear fashion over gestation, characterized by a period of rapid acceleration during a specific developmental window, rather than progressing uniformly across time.

We extracted the inflection point of the fitted sigmoid curve for each node, representing the gestational age at which the rate of increase in nodal strength is maximal. Across all 88 regions, inflection points ranged from 27.7 to 30.5 weeks, with a mean of 28.6 ± 0.44 weeks. When analyzed by hemisphere, both the left and right hemispheres exhibited similar but statistically distinct distributions of inflection points (28.5 ± 0.35 vs. 28.7 ± 0.50 weeks, respectively), with a paired t-test revealing a significant difference (p=0.00012). We also compared the slope of the sigmoid curve at the inflection point, quantified by the inverse of the parameter k in Equation (1), and found significantly steeper slopes in the left hemisphere compared to the right $\left(p=10^{-5}\right)$. However, within each hemisphere, there were no statistically significant differences in either the timing or the rate of nodal growth across different brain lobes. These results suggest that while hemispheric asymmetries in the dynamics of connectivity maturation do exist, particularly a slightly earlier and more rapid increase in the left hemisphere, the broader developmental trajectory remains well-synchronized across brain regions. The tight clustering of inflection points around 27.7–30.5 weeks likely marks a critical window of structural reorganization and accelerated white matter development during late gestation.

**Figure 6:**
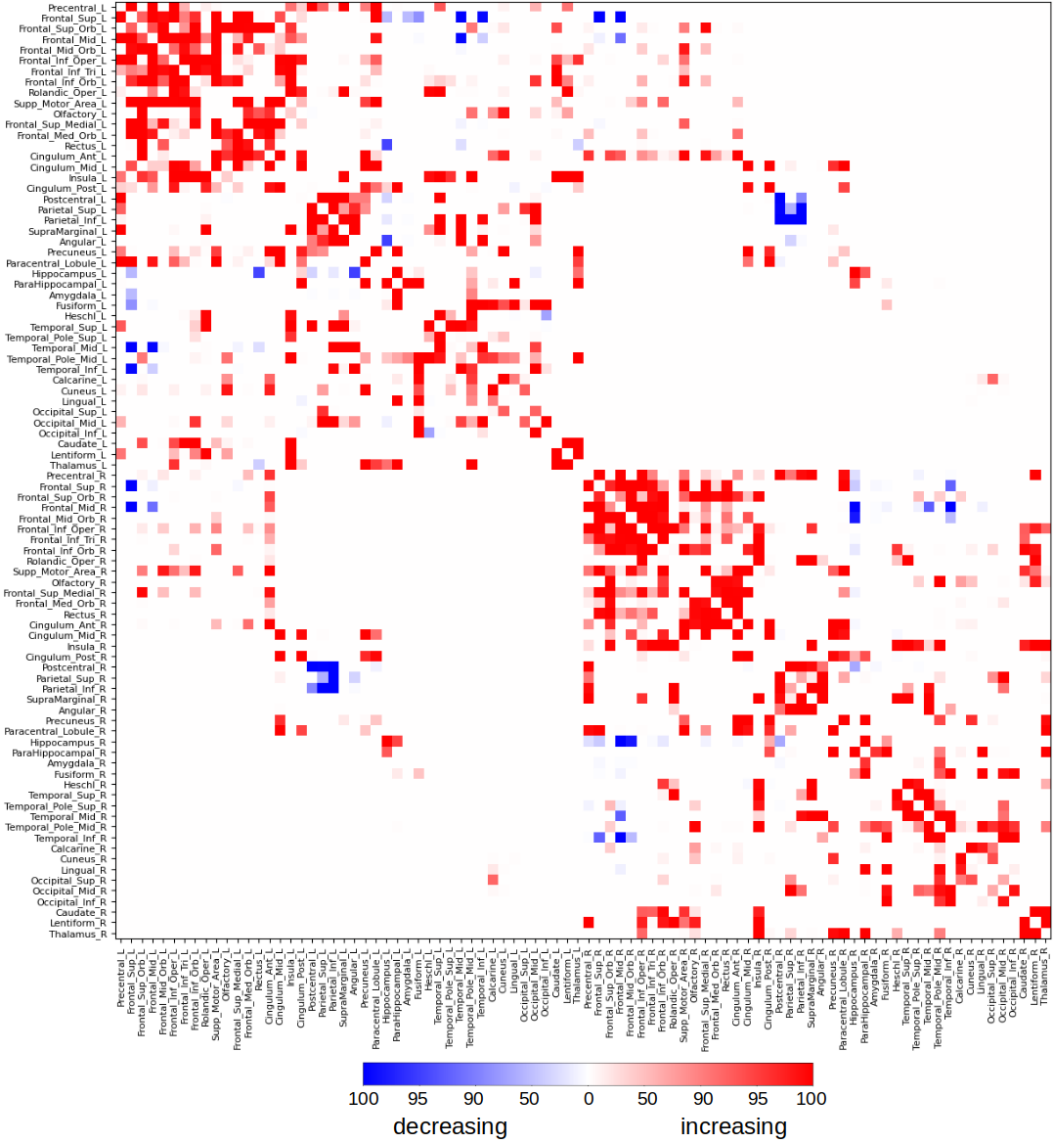
Change in the edge connection strength, i.e., the strength of connection between pairs of nodes, as a function of gestational age. We randomly selected 75% of the subjects and determined if there was a significant linear relation between the edge strength and gestational age. We repeated this 100 times for each edge. This figures shows the percentage of times the connection showed a significant increase (red) or decrease (blue) with gestational age.

We further investigated how the strength of individual connections between brain regions evolved over gestation by analyzing the temporal changes in edge strength. To assess the reproducibility of these developmental trends, we employed a robust resampling strategy. Specifically, for each network edge, we randomly selected 75% of the subjects without replacement and tested for a statistically significant linear association between edge strength and gestational age. This procedure was repeated 100 times per edge, and for each repetition, we recorded whether the edge exhibited a significant change. As shown in Figure 6, we summarized the results by calculating the percentage of repetitions in which each edge showed a significant association. To ensure high robustness, we retained only those edges that were significant in at least 95% of the repetitions. Based on this criterion, 162 edges demonstrated a consistent increase in strength over gestation, while 12 showed a consistent decrease. The strengthening edges encompassed both short-range and long-range connections, linking neighboring regions within lobes as well as distant regions across lobes and hemispheres. Notably, robust increases were observed in connections involving the anterior, middle, and posterior cingulate gyri, as well as in interhemispheric connections between the hippocampi. In contrast, the few connections that weakened with gestational age were primarily interhemispheric, including those linking the postcentral gyri, superior parietal lobules, and inferior parietal lobules in the parietal lobe, as well as the superior and medial frontal gyri in the frontal lobe. These findings suggest that while most structural connections strengthen during late gestation, a small subset of connections may be selectively pruned or refined as part of the maturation process.

#### 3.1.3 Network hubs across the gestation

Finally, we identified the hubs of the structural connectome across gestational ages, using three complementary centrality metrics: (1) Degree centrality, which reflects the total connection strength of a node and captures its local importance; (2) Betweenness centrality [100], which quantifies the extent to which a node lies on the shortest paths between other node pairs, indicating its role as a connector or bridge; and (3) Eigenvector centrality [101], which measures a node’s influence based on the importance of its neighbors and captures global network prominence. While degree centrality is inherently a local measure, both betweenness and eigenvector centrality capture global aspects of the network.

**Figure 7:**
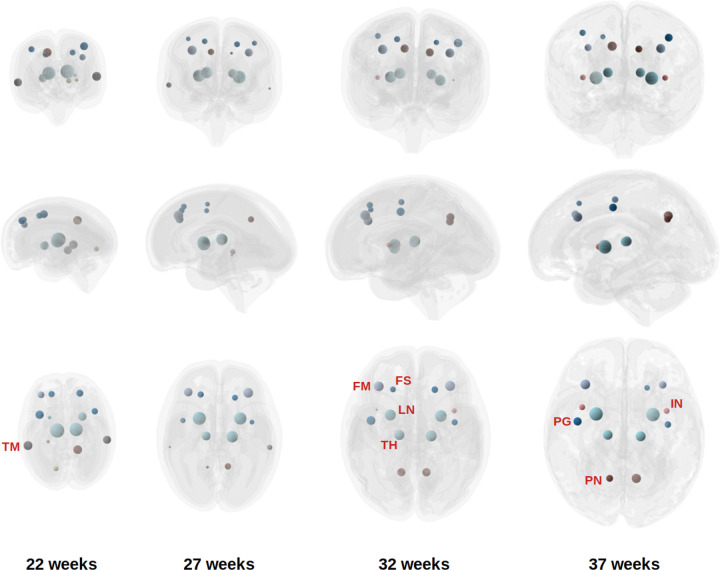
Network hubs identified on individual fetuses, averaged across all fetuses at each gestational week. The size of the sphere is proportional to the node strength for each hub. FS: Frontal superior gyrus; FM: Frontal middle gyrus; IN: Insula; LN: Lentiform; PN: Precuneus; PG: Precentral gyrus; TH: Thalamus; TM: Middle temporal gyrus.

Figure 7 illustrates the distribution of network hubs at four gestational ages, based on eigenvector centrality. Several brain regions consistently emerged as hubs across most gestational weeks (22–37 weeks), including the precentral gyrus, superior and middle frontal gyri, precuneus, lentiform, and thalamus. Some regions showed increasing prominence with gestational age, notably the supplementary motor area, medial portion of the superior frontal gyrus, and insula. Conversely, a few regions, such as the middle temporal gyrus and inferior temporal gyrus, exhibited a decline in hub status over time, although they remained strongly connected.

Overall, there was substantial concordance among the three centrality measures, though some differences were observed. When defined by degree and betweenness centrality, the middle frontal gyrus and inferior temporal gyrus appeared less prominent, while the angular gyrus, fusiform gyrus, superior parietal lobule, and inferior parietal lobule emerged as more prominent hubs under these criteria.

Our findings broadly align with previous studies on structural connectome hubs in the human brain. In adults, commonly reported hub regions include the precuneus, superior frontal and superior parietal cortices, anterior and posterior cingulate cortex, insular cortex, temporal cortex, lentiform/putamen, thalamus, and hippocampus [102–108]. Several studies have also examined hub development in neonates and infants, often using functional MRI–based connectomes [57,109–111]. Reported structural hubs in neonates include the dorsal and medial frontal cortex, parietal cortex, precuneus, hippocampus, insula, and anterior cingulate cortex [7, 112, 113]. Functional hubs during the fetal and neonatal periods have been reported to include the cingulate cortex, precentral and postcentral gyri, superior parietal lobule, cerebellum, angular gyrus, fusiform gyrus, insula, and association cortices near the fusiform facial area and Wernicke’s area [55, 57, 109, 111, 114–117]. The considerable overlap between hubs identified in our study and those reported in the literature lends support to the biological plausibility and generalizability of our findings. Notably, however, we did not identify the hippocampus or the cingulate cortex as major hubs, despite their frequent appearance as hubs in prior studies. These discrepancies may reflect differences in imaging modality, gestational age range, or methodological approach, including the definition of hub metrics and tractography techniques. In fetal imaging, in particular, there is a lack of standard computational methods, and difference in definitions of the extent of different cortical regions can lead to different results in determination of structural connectivity hubs.

### 3.2 Age-specific Connectome Templates

#### 3.2.1 Comparison of different template reconstruction methods

**Figure 8:**
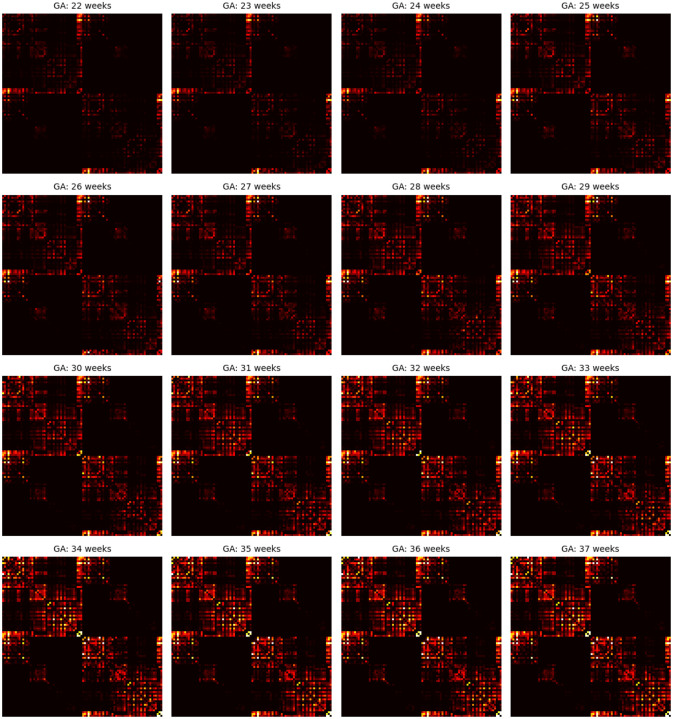
Connectome templates computed for every gestational age between 22 and 37 weeks with the connectome aggregation method proposed in Section 2.4.1.

**Figure 9:**
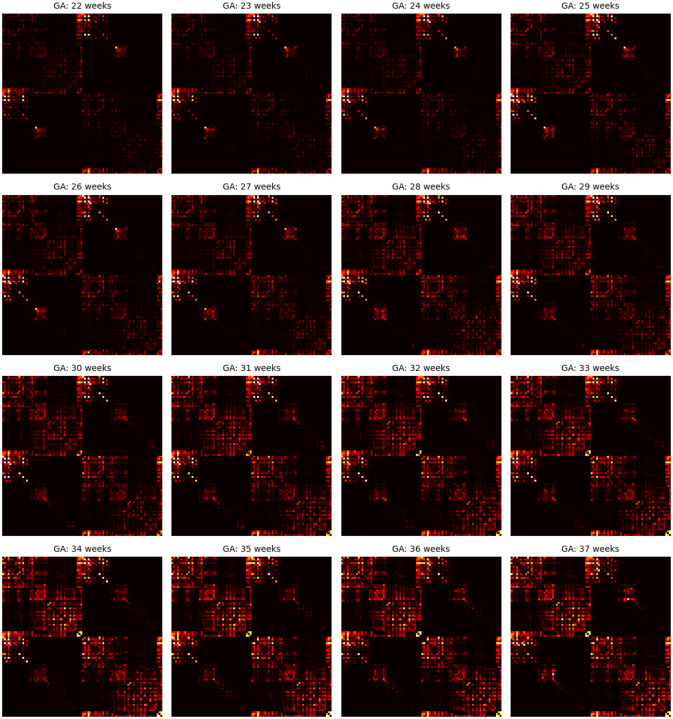
Connectome templates computed for every gestational week via data averaging in the image space described in Section 2.4.2.

Figure 8 presents the age-specific connectome templates for gestational weeks 22 to 37, generated using the proposed connectome aggregation method described in Section 2.4.1. For comparison, Figure 9 displays the corresponding templates constructed via image–space averaging, as detailed in Section 2.4.2. Both approaches yield connectomes that demonstrate a consistent increase in connection strength over gestation – within individual brain lobes, across different lobes, and between hemispheres. While the overall topological trends are broadly similar between the two methods, the templates derived from image–space averaging tend to show stronger inter-hemispheric connections. This is likely due to the spatial smoothing effect inherent in voxel-wise averaging across multiple subjects.

To quantitatively evaluate the performance of these methods, we compared the resulting templates to the individual connectomes of age-matched fetuses. Table 1 summarizes this comparison, which also includes the baseline method of distance-preserved averaging of connectomes [90]. The results for consensusbased thresholding were largely similar to distance-preserved averaging and, hence, are omitted from this table.

For each template, we measured the difference in several key graph–theoretical metrics including global efficiency (GE), local efficiency (LE), characteristic path length (CPL), and total network strength, between the template and the corresponding individual connectomes. Across all metrics, the connectome aggregation method yielded templates that more closely resembled individual subject connectomes. Figure 4 shows a visual representation of these metrics.

We also evaluated the ability of each connectome template to capture age-related patterns using classification experiments. First, a nearest-neighbor classifier based on graph edit distance (GED) was used to predict the gestational age. Templates constructed via connectome aggregation achieved the lowest prediction error (0.98 ± 0.95 weeks), substantially outperforming both image–space averaging and element–wise averaging, which yielded errors greater than 2 weeks. Next, we implemented a more sophisticated classification framework based on the method in [98], described in Section 2.4. This method achieved an error of 0.90 weeks with the connectome aggregation templates, outperforming the other two methods (1.44 and 1.46 weeks, respectively). All results were validated using ten-fold cross-validation.

Finally, we assessed how well each method preserved the location of high-centrality nodes (i.e., hubs). For each gestational age, we identified the top 10 hubs in individual fetuses and their corresponding templates, and calculated the percentage of overlap. As shown in Table 1, the connectome aggregation–based templates had the highest agreement with the individual–level hubs (83% overlap), followed by image–space averaging (80%), and distance-preserved averaging (72%). These results indicate that connectome aggregation not only preserves overall network topology but also retains region–specific nodal prominence better than the other methods.

#### 3.2.2 Analysis of spatiotemporal trends based on the connectome templates

**Figure 10:**
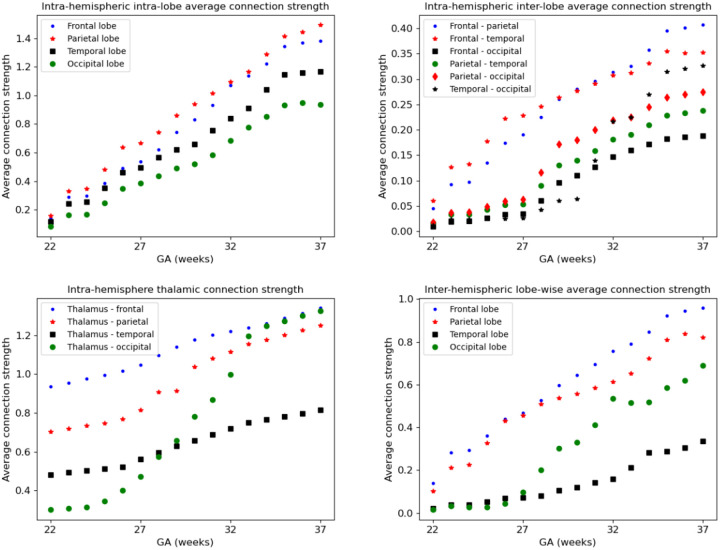
Development of structural connectivity across different brain regions computed using the connectome templates constructed with connectome aggregation method.

As summarized in Table 1 and illustrated in Figure 4, the connectome templates reconstructed using the connectome aggregation method outperformed those generated by alternative approaches in preserving key network characteristics. These high–fidelity templates were therefore used to analyze the developmental evolution of structural connectivity. Figure 10 presents four sets of plots illustrating the temporal dynamics of connectivity across different brain regions computed using these templates. Consistent with these visualizations and the connectome matrices shown in Figure 8, the fetal brain undergoes marked and progressive changes in structural connectivity between 22 and 37 gestational weeks.

The developmental trajectories are largely smooth and continuous, without any abrupt transitions. Nonetheless, for interpretability, we divided this interval into five approximately equal sub-periods, each capturing key developmental transitions during this critical stage of neurodevelopment.

##### Stage 1: 21–23 gestational weeks.

At this early stage, global connectivity remains weak. Subcortical–cortical connections begin to emerge, particularly those linking the thalamus and lentiform nuclei to the developing frontal and parietal cortices. The corpus callosum is present but immature. Anatomically, the genu (the anterior portion of the corpus callosum) is among the first to form, allowing interhemispheric connection between homologous frontal areas. Likewise, the body of the corpus callosum begins to connect bilateral frontal and parietal lobes. Cortico–cortical connectivity within and between the lobes remains sparse, and although tractography recovers many intra–hemispheric association streamlines, their estimated weights based on fiber bundle capacity are negligible, reflecting immature or still-developing pathways.

##### Stage 2: 24–26 gestational weeks.

This stage marks an increase in the strength of association pathways. Notably, intra-hemispheric connections between frontal and parietal lobes and between frontal and temporal lobes experience strengthening. The splenium of the corpus callosum, which connects the occipital lobes across hemispheres, begins to mature during this window, though these interhemispheric occipital connections remain weaker than their frontal and parietal counterparts. Thalamocortical projections to early visual areas such as the calcarine cortex, cuneus, and lingual gyrus also emerge, marking the onset of organized input to the occipital lobe. This can be seen in the plots as the early rise in thalamus–occipital connectivity.

##### Stage 3: 27–29 gestational weeks.

This period is characterized by a consistent increase in the strength of nearly all connections, with several connections showing more pronounced acceleration. These include inter-hemispheric connections between the occipital lobes as well as the connections of occipital lobes to the thalami. Long-range intra-hemispheric association pathways also become more prominent, particularly those linking the occipital lobe to temporal and parietal regions. These maturing pathways form the anatomical substrate for higher cognitive and sensory integration processes that will support functions such as language, attention, and memory. Thalamocortical pathways continue to refine, particularly those targeting sensory and association cortices, contributing to the emerging coordination of large-scale brain networks.

##### Stage 4: 30–32 gestational weeks.

Connectivity continues to strengthen globally, but several regional developments stand out. Interhemispheric temporal lobe connections, while still on average less strong than those of the frontal and parietal lobes, show signs of growth. Interhemispheric integration in the occipital lobe accelerates further as the splenium of the corpus callosum matures. Inter-lobe connections between the occipital and temporal lobes show a highly accelerated pace, much faster than all other intra-hemispheric inter-lobe connections at this stage, suggesting growing interdependence between visual and auditory/language processing areas. The insula, a key multisensory and salience–processing hub, exhibits increasing connectivity with multiple lobes, indicating its emerging role in integrating sensory, emotional, and cognitive information.

##### Stage 5: 33–37 gestational weeks.

By late gestation, most major cortico-cortical association pathways – including those linking the temporal, occipital, and parietal lobes – are well established. Connectivity within the limbic system, particularly involving the amygdala and hippocampus, strengthens significantly, especially in their projections to the cingulate and temporal cortices. While connection strength continues to increase both within and across lobes and hemispheres, the rate of change slows down in some pathways. This may suggest that the overall scaffold of the neonatal connectome is nearing completion by term-equivalent age. Postnatal development will likely involve fine-tuning processes such as myelination, pruning, and synaptic remodeling, building upon this robust structural backbone.

### 3.3 Reproducibility analysis

We evaluated the reproducibility of the reconstructed structural connectomes by assessing their consistency across independent subsets of dMRI measurements. The dMRI data for each fetus consisted of two shells: 46 measurements at b=400 and 80 measurements at b=1000. For each shell, we divided the measurements into two non-overlapping halves and reconstructed a separate connectome from each subset. Denoting the connectomes reconstructed for fetus i from the two subsets as $C_{i}^{A}$ and $C_{i}^{B}$, we computed the graph edit distance between them to quantify intra-subject variability. As a reference, we also computed inter-subject GEDs by comparing $C_{i}^{A}$ to $C_{j}^{A}$ and $C_{i}^{B}$ to $C_{j}^{B}$ for all i≠j. Intra-subject GEDs were significantly lower than inter-subject GEDs, with mean values of 5038 ± 1745 and 13619 ± 2603, respectively. For all 198 fetuses, the two connectomes derived from the independent subsets of their data were more similar to each other than to the connectomes of any other subject. This result demonstrates strong reproducibility of individual–specific structural connectivity profiles.

In addition, we computed several graph-theoretical metrics – SWI, CPL, GE, LE, CC – from each connectome derived from the two measurement subsets. We then fitted linear models to assess how each metric varied with gestational age, separately for each subset. The resulting regression models showed no significant differences in slope or intercept across the two data halves for any metric p>0.05, further confirming the robustness and reproducibility of the estimated connectomes and the derived network properties.

## 4 Conclusions

To the best of our knowledge, this study represents the largest investigation of the structural connectivity of fetal brain based on diffusion MRI. Analysis of connectivity metrics including CPL, GE, CC, and LE revealed a monotonic increase in modular integration and segregation of the structural brain networks in this period. Our results also showed a small-world configuration, persistent across the studied period between 22 and 37 gestational weeks. Our findings showed that the connection strength for all 88 nodes considered in this analysis increased as a function of gestational age. The results of a sigmoid function fit to the data revealed inflection points, marking the fastest increase in connection strength, between 27.7 and 30.5 weeks. This time window overlaps with the period of 29–31 gestational weeks that has been observed to be important in a recent study of functional connectivity of the fetal brain [87]. That study found that the strength of thalamocortical and cortico–cortical functional connectivity increased sharply between 29 and 31 gestational weeks. Additionally, other studies on white matter maturation in terms of FA and MD have shown critical windows that are also similar [44, 118]. For instance, an analysis of 34 different white matter tracts has shown that both FA and MD display a turning point between 27 and 32 weeks [118]. Hence, our results further confirm the importance of this time window to the formation of brain networks in utero.

A technical contribution of this work was the new methods for constructing connectome templates to represent the typical development of structural networks in the fetal period. Our evaluations showed that our novel approach based on aggregation of the individual connectomes performed better another approach based on precise spatiotemporal alignement and averaging of data in the image domain. Our analysis showed that connectome-wise averaging led to connectome templates that represented connectomes of similar-age fetuses very closely in terms of connectivity metrics and hubs. Averaging in the image space, though a sophisticated approach, resulted in connectomes that deviated from individual fetuses. This deviation is likely due to preferential weakening of certain connections and strengthening of other connections as a result of data averaging in the image space as implemented in this project. Our approach to data averaging in the image space, described in Section 2.4.2 and Figure 1, is largely similar to the approaches followed by many prior studies on fetal brain imaging [43,94,118,119]. Therefore, our findings suggest that these widely-used approaches to constructing fetal brain templates cannot preserve the features of individual fetal brain connectome, although they may be suitable for other purposes such as studying distinct brain regions or local development of brain structure and microstructure.

Using the connectome templates generated via connectome aggregation, we characterized spatiotemporal trends in fetal brain connectivity between 22 and 37 gestational weeks. Although the developmental trajectory appeared largely continuous, we divided this period into five developmental sub-phases to highlight the key transitions in structural maturation. Around 22 weeks, thalamocortical connections are relatively much stronger than cortical-cortical connections, which are still immature both within and between hemispheres. By 37 weeks, on the other hand, dense and symmetric connections are observed within each lobe, across lobes, and between homologous regions across hemispheres. Each stage of development showed specific patterns of accelerated growth in particular connections, such as thalamocortical visual pathways, intra– and inter–hemispheric occipital–temporal associations, and insular hub emergence, reflecting the sequential establishment of large–scale functional systems during gestation.

Although the reproducibility of tractography and quantitative connectomics remains a topic of ongoing research, our reproducibility analysis yielded promising results. Connectomes reconstructed from two non-overlapping subsets of dMRI measurements for each fetus were substantially more similar to each other than to those of any other subject, demonstrating strong individual specificity. Bootstrapped assessments of nodal and edge–wise connectivity further confirmed statistically robust and reproducible patterns, as illustrated in Figures 5 and 6. Additionally, the ability of the age-specific connectome templates to predict gestational age with an average error of less than one week suggests that the structural connectome captures meaningful and temporally specific developmental signatures. Collectively, these findings indicate that, despite inherent limitations in fetal dMRI and tractography, current methodologies are capable of capturing individualized profiles of fetal brain connectivity. As such, structural connectomics may serve as a valuable framework for investigating normative and atypical patterns of prenatal brain development.

Despite general agreement with existing literature, some differences between our findings and prior studies are notable. For instance, the SWI values observed in our study were lower and more stable than those reported previously, where values substantially greater than one and sharp increases with gestational age have been described [55, 58, 60, 120–122]. Similarly, although our hub analysis identified regions in agreement with previous structural and functional studies including the precuneus, thalamus, lentiform, and medial frontal cortex, we did not consistently identify the hippocampus or cingulate cortex as major hubs. These discrepancies may stem from several factors. For example, different definitions of the small–world index exist, and not all prior studies clearly report their chosen definition [123–125]. More importantly, many earlier works have used deterministic tractography and surrogate connectivity measures such as streamline count or FA [55, 58, 60, 126, 127], whereas in this study we have used anatomically constrained tractography tailored to fetal anatomy [78] and a biologically grounded measure of connection weight based on fiber bundle capacity [9, 85]. Finally, the size and quality of the data included in this study far exceed those used in prior fetal studies, allowing for more robust and generalizable conclusions. Therefore, we believe our findings offer new insights that may help reinterpret the results of earlier studies on this topic.

## Figures and Tables

**Table 1:** Quantitative comparison of the connectome templates computed using different methods. The first four rows show the difference in the connectivity metrics between the individual subjects and their age–matched connectome templates. The next two rows show the accuracy of predicting the age of an individual fetus based on the similarity of his/her connectome with the age–specific connectome templates. More specifically, these two rows show the error (in weeks) in the predicted age of individual fetuses based on the similarity to the connectome templates. The last row shows the overlap of the 10 top hubs between each fetus and its age–matched connectome.

	Connectome aggregation method	Data averaging in the image space	Distance-preserved averaging [90]
GE	**0**.**240** ± **0**.**207**	0.375 ± 0.422	0.352 ± 0.228
LE	**0**.**039** ± **0**.**029**	0.058 ± 0.036	0.040 ± 0.037
CPL	**246** ± **228**	254 ± 214	256 ± 126
Network strength	**6**.**63** ± **6**.**21**	8.05 ± 6.83	8.38 ± 6.40
Age prediction error (in weeks), computed using on a nearest-neighbor classifier based on graph edit distance (GED)	**0**.**98** ± **0**.**95**	2.01 ± 1.39	2.77 ± 1.45
Age prediction error (in weeks), computed using a support vector machine classifier as in [98]	**0**.**90** ± **0**.**81**	1.44 ± 1.37	1.46 ± 1.85
Hub overlap	**0**.**83** ± **0**.**08**	0.80 ± 0.06	0.72 ± 0.07
